# Relationship between Structure and Conformational Change of the Vitamin D Receptor Ligand Binding Domain in 1α,25-Dihydroxyvitamin D3 Signaling

**DOI:** 10.3390/molecules201119713

**Published:** 2015-11-18

**Authors:** Lin-Yan Wan, Yan-Qiong Zhang, Meng-Di Chen, You-Qin Du, Chang-Bai Liu, Jiang-Feng Wu

**Affiliations:** 1 Medical College, China Three Gorges University, 8 Daxue Road, Xiling District, Yichang 443002, China; wanlinyan0224@163.com (L.-Y.W.); zhangyanqiong@ctgu.edu.cn (Y.-Q.Z.); 15572753727@163.com (M.-D.C.); youqindu@126.com (Y.-Q.D.); 2 Hubei Key Laboratory of Tumor Microenvironment and Immunotherapy, China Three Gorges University, 8 Daxue Road, Xiling District, Yichang 443002, China

**Keywords:** ligand binding domain, vitamin D receptor, structure basis, functional domains, conformational change

## Abstract

Vitamin D Receptor (VDR) belongs to the nuclear receptor (NR) superfamily. Whereas the structure of the ligand binding domain (LBD) of VDR has been determined in great detail, the role of its amino acid residues in stabilizing the structure and ligand triggering conformational change is still under debate. There are 13 α-helices and one β-sheet in the VDR LBD and they form a three-layer sandwich structure stabilized by 10 residues. Thirty-six amino acid residues line the ligand binding pocket (LBP) and six of these residues have hydrogen-bonds linking with the ligand. In 1α,25-dihydroxyvitamin D_3_ signaling, H3 and H12 play an important role in the course of conformational change resulting in the provision of interfaces for dimerization, coactivator (CoA), corepressor (CoR), and hTAF_II_ 28. In this paper we provide a detailed description of the amino acid residues stabilizing the structure and taking part in conformational change of VDR LBD according to functional domains.

## 1. Introduction

Vitamin D Receptor (VDR) belongs to the nuclear receptor (NR) superfamily and consists of 427 amino acid residues. It is composed of four major functional domains: A/B domain, a highly variable amino-terminal; C domain (DNA binding domain, DBD), a highly conserved zinc finger DNA-binding domain; D domain, a hinge domain; and E domain (Ligand Binding Domain or LBD). The VDR LBD plays an important role in facilitating ligand binding, nuclear localization, dimerization, and interaction with coactivator (CoA) and corepressor (CoR) proteins [1].

Like other NRs, VDR LBD includes 13 α-helical (H) structures, of which the H12 contain a short activation function-2 (AF-2) domain. It is folded into a three-layered, antiparallel α-helical sandwich that creates a ligand binding pocket (LBP). VDR LBD presents a Ω loop between H2 and H3, and its conformation is flexible [2,3].

It is a central step in 1α, 25-dihydroxyvitamin D_3_ (1,25(OH)**_2_**D**_3_**) signaling that ligands trigger the conformational change of VDR LBD resulting in formation of some interfaces for dimerization, CoAs and CoRs. It is unclear how the VDR LBP “adapts” to so many various vitamin D analogues and how the flexible structure of VDR LBD after different ligand binding produces the different effects. It easily causes misunderstandings about the α-helical structures of VDR LBD and difficulties for readers and researchers, so this review attempts to understand the relationship of structure and function of VDR LBD.

## 2. Structure of VDR LBD

**Figure 1:**
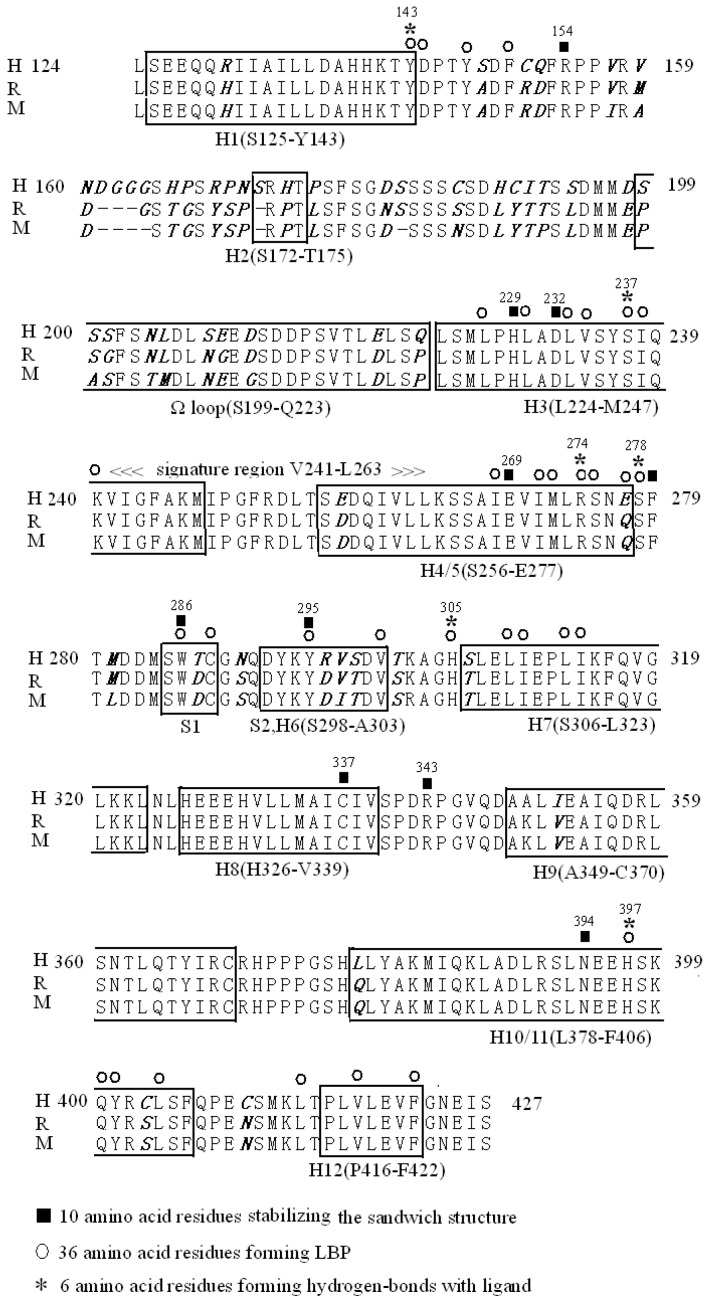
Alignment of a portion of the VDR LBD of human, rat and mouse. The amino acid sequence of VDR LBD extends from residues 142 to 427 in humans, to 423 in rats, and to 422 in mice. The closed boxes indicate the position of the 12 α-helices and the 2 β-strands, forming a β-sheet and H3n (SVTLE) between residues 216–220. Ten solid bars above the VDR sequence correspond to the amino acid residues in the VDR LBD which stabilize the sandwich structure and 36 closed rings above the VDR sequence corresponding to amino acid residues in the VDR LBD which form LBP. Among them, six asterisks represent polar amino acid residues within a 3 Å distance from 1,25(OH)_2_D_3_ which form hydrogen-bonds with the ligand. Only VDR LBD including the Ω loop of 25 residues from S199 to Q223 cannot exist in other NRs.

VDR LBD exhibits a high functional domain sequence homology in human, rat, and mouse orthologues, except for the amino acid residues between H1 and H3. The VDR LBD of humans extends from residues 124 to 427 and includes a loop of 25 residues from S199 to Q223, with a Ω loop structure between H2 and H3. Although the Ω loop is not specific to VDR, all NRs have a Ω loop but VDR has an additional insertion domain between H2 and H3. There are 10 residues that have a greater role in stabilizing the structure of VDR LBD [2] and 36 residues taking part in forming LBP, of which six residues link to the ligand by hydroxyl groups [4] (Figure 1). There are also other residues taking part in forming interfaces to provide the RXR, CoAs, and CoRs [5].

**Figure 2:**
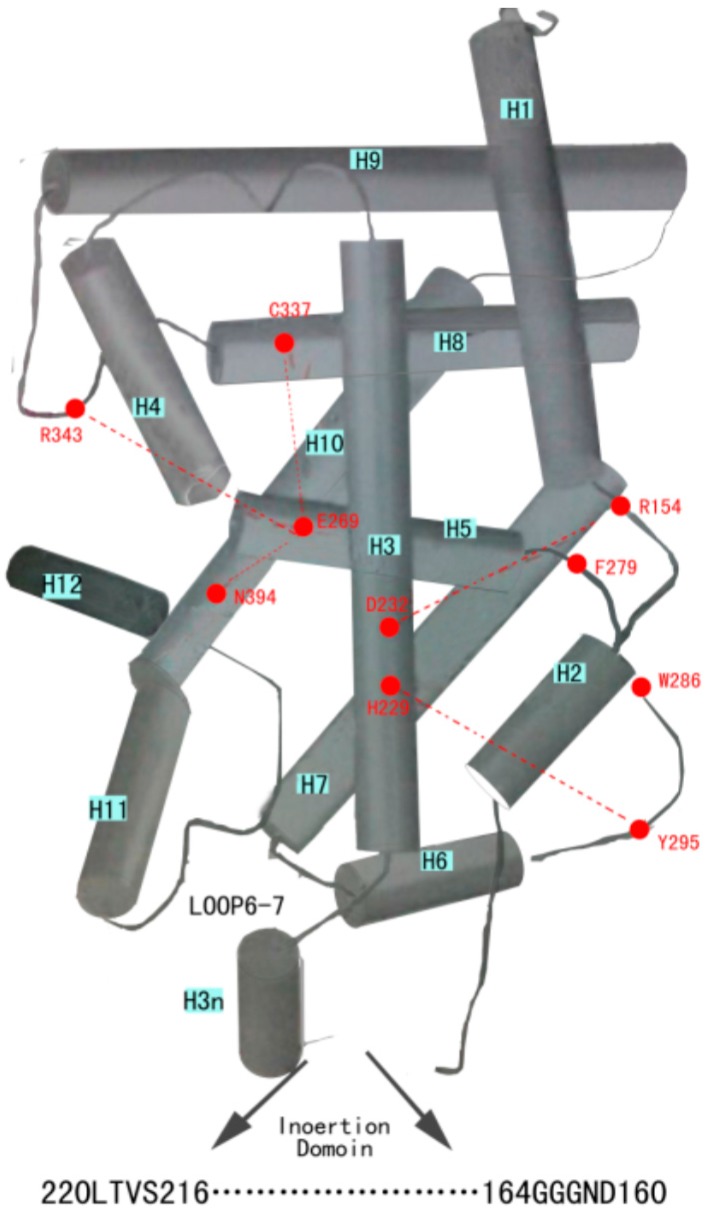
Important amino acid residues. There are 10 important amino acid residues which have a greater role in stabilizing the sandwich structure of VDR LBD with no-ligand. There is a center crossing of H3 with H5. D232 and H229 of H3 form hydrogen bonds with R154 (just behind H1) and Y295 (β-sheet just in front of H6) respectively. E269 (H5) is connected by hydrogen bond to C337 (H8), R343 (coil between H8 and H9), and N394 (H10). The β-sheet between H5 and H6 originates from a strong hydrophobic interaction formed by F279 (β-sheet just behind H5) and W286 (β-sheet in front of H6).

With the notation to Figure 1 LBD consists of 13 α-helices and two β-strands forming a β-sheet, and it has already been successfully crystallized. The alignment is the same as that reported by Moras’ group [3] and Yamamoto’s group [6] but is different from the alignment reported by Norman’s group [7] at the position of H1. These helices are folded into a three-layered, antiparallel α-helical sandwich to form a hydrophobic binding pocket for the hormone in the three-dimensional structure. The first layer is H1 to H4, the second is H5 to H9, and the third is H10 and H11. H3n and H12 project outside to LBD in unligand-binding [3]. The helical sandwich structure with three layers of α-helices surrounds the hydrophobic LBP [2,3]. In fact, this helical sandwich crystal structure of VDR LBD is the basis of engineering a VDR LBD without a flexible insertion domain with reference to Figure 2 by removing the residues 165-215 of VDR, thereby leaving 29 residues to connect the H1 and H3 (there are 80 residues from D144 to Q223 between H1 and H3). Up until now, no biological significance is found for the particular region of VDR LBD between H1 and H3 with a poorly conserved large insertion domain [2].

With reference to Figure 2 there are 10 amino acid residues that have a greater role in stabilizing the sandwich structure and provide the basis for LBD [2]. There are two pairs of these residues that form hydrogen bonds to link H3, H1, and H6 together. D232 (H3) forms three hydrogen bonds with R154 (just behind H1) binding these areas strongly together; the substitution of D232A destabilizes the full-length receptor structure and causes formation of a nonfunctional receptor protein. H229 (H3) and Y295 (β-sheet just in front of H6) are bound together by a hydrogen bond connecting H3; the β-sheet stabilizes the receptor structure because replacing any of these two amino acid residues with alanine leads to an unstable receptor protein that is biologically inactive [8]. There are another two pairs of residues that form hydrogen bonds to link H5, H8, H9 and H10 together. E269 (H5) is connected by a hydrogen bond to C337 (H8), R343 (coil between H8 and H9), and N394 (H10), and connects four helices with each other. In fact, E269 does not interact directly with calcitriol and its importance is in the stabilization of the interior of the VDR structure because substituting E269 with alanine produces a significant decrease in the stability of full-length VDR LBD and the biological activity of the receptor with ligands. The β-sheet between H5 and H6 has its origin from a strong hydrophobic interaction formed by F279 (β-sheet just behind H5) and W286 (β-sheet in front of H6) and the substitution F279A strongly decreases the stability of the receptor. Substitutions W286A and W286F completely abolish binding of calcitriol and this dramatically decreases the ligand binding affinity and stability and is attributable to a breakdown of the hydrophobic interaction between W286 and F279 that destabilizes the receptor structure. In hereditary vitamin D-resistant rickets (HVDRR), isoleucine is replaced by threonine at amino acid 268 (I268T) in exon 7 of the VDR gene [9,10].

## 3. The Ligand Binding Pocket of the Vitamin D Receptor

Several approaches can be used to determine the structure of the LBP of the VDR, such as statistical coupling analysis (SCA), X-ray crystal structure analysis, total alanine scanning mutational analysis (ASMA), two-dimensional ASMA (2D-ASMA), ligand-mediated allosteric network analysis, and the *ab initio* fragment molecular orbital (FMO) method at the Møller–Plesset second-order perturbation (MP2) (that is FMO-MP2 method) [11,12]. Although several approaches can be used to determine the structure, only NMR and X-ray crystallography provide 3D structure at atomic resolution, the other methods can only probe the LBP.

The VDR-LBP is made up of 36 amino acid residues (two alanine residues) that lie mainly in H3, H5, H6, H7, H11, and H12 [4]. There are seven residues (L227, L230, L233, V234, S237, I238, and K240) in H3, 16 residues (I268, I271, M272, R274, S275, E277, S278, W286, C288, Y295, V300, H305, L309, I310, L313, and I314) from H5 to H7, and seven residues (H397, Q400, Y401, L404, L414, V418, and F422) in H11 and H12. There are also four residues (Y143, D144, Y147, and F150) just behind H1. There are 14 hydrophilic residues, six of these residues (Y143, S237, R274, S278, H305, and H397) are hydrogen-bonding residues and the other eight hydrophilic residues are D144, Y147, K240, S275, E277, S295, Q400, and Y401. There are 20 hydrophobic residues, including seven leucine residues (L227, L230, L233, L309, L313, L404, and L414), five isoleucine residues (I238, I268, I271, I310, and I314), three valine residues (V234, V300, and V418) and five other hydrophobic residues (F150, M272, W286, C288, and F422) [13].

According to the distance to 1,25(OH)**_2_**D**_3_**, these amino acid residues are divided into four classes [4]. First, there are six polar residues within a 3 Å distance from 1,25(OH)_2_D_3_ that form three pairs of residues that link to three hydroxyl groups (1-OH, 3-OH, and 25-OH) of 1,25(OH)**_2_**D**_3_** by hydrogen-bonds. Each of the three hydroxyl groups forms a pincer-type hydrogen bond with a pair of residues [14]. They are S237 (H3) and R274 (H5) for 1 α-OH, Y143 (H1) and S278 (just behind H5) for 3β-OH, and H305 (just in front of H7), and H397 (H11) for 25-OH. These six residues are important for conformation change in ligand-binding, *i.e.*, the substitutions of H305A and H397A can clearly shift the conformation of full-length VDR to the non-agonistic direction [4,15].

Hydrogen-bonding interactions between R274 and 1-OH, Y143, and 3-OH, H397 and 25-OH, are more important than those of S237, S278, H305 respectively. For example, in gene activation assays, VDR constructs with the single mutation, Y143F, and the double mutation Y143F/S278A, but not the single mutation, S278A, require higher doses of 1,25(OH)**_2_**D**_3_** for half-maximal response. This suggests that Y143 residue is more important for receptor function than residue S278 [16]. In fact, the Van der Waals interaction energies of these six hydrogen-bonding residues are similar, and the substantial differences between the overall interaction energies are due to electrostatic interactions. H397 plays a key role in maintaining the stability of the active conformation by forming a π-cation type interaction with F422 (H12). The function of the 1-OH or the 25-OH may be more important than the 3-OH of ligand because of the deletion of 1-OH or 25-OH leads to a remarkable decrease in binding (1/1000), whereas the deletion of the 3-OH is less effective (1/20) [15].

Second, there are 13 residues at approximately a 3–4 Å distance from the ligands that have hydrophobic interaction with the ligand. Of these nine residues, Y147, L227, L230, L233, I271, W286, Y295, L309, and V418 are essential, and they interact with other residues on different secondary structural units to finish ligand-mediated protein folding. Three of these residues, V234, S275, and V300 are less important. C288 can reversibly bind to 1,25(OH)**_2_**D**_3_**, but irreversibly bind to 1,25-dihydroxy- vitamin D_3_-3β-bromoacetate (1,25(OH)**_2_**D**_3_**-3-BE). In fact, C288A destabilizes the VDR LBP, resulting in a severe decrease of transcriptional activity due to its breakdown of Van Der Waals linking in Y147, F150, S278, and Y295 to C288 [15,17].

Third, there are eight residues approximately 4–5 Å distance from the ligand. F150, I268, L401, L404, L414, and F422 are important for protein folding. M272 and L313 are less important. Finally, there are seven residues more than a 5 Å distance away from the ligand. Among these, D144 and I238 are important for folding. K240, E277, I310, I314, and Q400 are not important [4].

Electrostatic interactions are the major determinant of the ligand-binding activity and ligand recognition specificity, and Van Der Waal’s interactions are important for protein folding, and in turn, for cofactor binding. When these contact sites are occupied, the ligand is stabilized within the LBP by several minor interactions that depend on the structure and flexibility of the ligand side chain, and thus, may differ between different ligands, such as calcitriol and lithocholic acid [11,18,19]. Calcitriol and plenty of its analogs are very flexible due to their open B-ring structure. Thus, they easily fit into the LBP with conformational changes, then reach the potential contact sites. The flexibility probably makes them to have a distinct number of weaker interactions with the receptor because bent and straight side chains of ligand can interact with a variety of amino acid residues of the receptor [20].

## 4. The Activation Domain of VDR LBD

Ligand binding creates a transactivation surface or platform for the binding of p160 CoA proteins and also H4-5 not just H3 and H12 contribute to this transactivation platform for the VDR. Thus, the activation domain of VDR LBD consists mainly of two regions, one of which is related with H12 and another is related with H3. A part of CoA interaction surface is made up of some candidate domains near the AF-2 core. These candidate domains contain the exposed residues on the surfaces of H3 and H4, the loop between H11 and H12 as well as the region between H1 and H3, comprising the Ω loop. In fact, the A/B domain of the VDR consists of only 20 amino acids and the deletion of these residues has no impact on VDR-mediated transcription. Thus, the VDR lacks an AF-1 transactivation function in the NH2-terminus. Recently, a unique activation function 2a (AF-2a) domain was identified in the mid-region of the ER LBD and whether it exists in the VDR is worthy of future discussion [21].

### 4.1. The First Activation Domain in the VDR LBD

The α-helix carboxy-terminal of VDR-LBD, H12, includes 11 amino acid residues (residues 417–427), which are LVLEVFGNEIS, and removal of the entire H12 (including the AF-2 domain) does not break down the structure of VDR; however, the deletion of H12 causes formation of an unstable receptor with all ligands. The last five amino acid residues (GNEIS) of H12 (residues 423–427) are often called the AF-2 domain, but do not significantly contribute to ligand binding. The function of the AF-2 domain is related to mediating signal activation or repression [22].

Alanine-scanning mutagenesis shows amino acid residues 417–422 are critical for mediating the activity of the natural hormone. Specifically, mutation of amino acid residues L417, E420, and F422 nearly completely abolish the activity of the VDR. C-terminal truncations and point mutations of VDR identified amino acid phenylalanine 422 (F422) is of central importance for the high affinity ligand binding conformation of VDR, and this amino acid may directly contact the ligand and correct the positioning of H12 [23].

Ligand-triggered protein–protein interactions are the central molecular events of nuclear 1,25(OH)**_2_**D**_3_** signaling, and there is only one agonistic conformation of the VDR LBD. In case of the agonist, H397 (H11) contacts the OH-group at the C25-hydroxyl group of the ligand, and is supported by an additional, less important hydrogen bond with H305 (just in front of H7). Then, H397 contacts F422 (H12) and forms the H397–F422 bridge that may stabilize H12 that forms the “lid” folding down onto the surface of the LBP, where it is thought to close off the hydrophobic LBP and project its inner hydrophobic surface towards the bound hormone and the other surfaces are packed against H3, 5, and 11. At the same time, the bridge H397–F422 lays at a distance of 19 Å between a negatively charged E420 on the surface of H12 and a positively charged K246 on the surface of H3. This charge clamp structure provides optimal positioning for contacting the LXXLL motif of the VDR interaction box of CoA proteins [24].

The extended side chains of the antagonist, disturbing the interaction between H397 and F422, destabilize the agonistic conformation of H12, and consequently, the positioning of E420. This decreases the affinity of CoAs or even makes interaction impossible [25].

In the inverse agonistic conformation, H12 does not move in comparison to the apo-form of the VDR, because of which, the receptor is unable to contact CoA proteins; instead, it recruits CoR proteins and mediates super-repression. Amino acid F422 (H12) has a critical role in this process and the result is that CoR proteins do not dissociate from the receptor and block the binding of a CoA protein. Gemini, a sensor for cell-specific CoA/CoR ratios, which is a 1,25(OH)**_2_**D**_3_** analogue composed of two characteristic side chains, can stabilize the VDR into a silent conformation, in which it is unable to contribute to CoA interaction because of the important role of F422 resulting in reposition of H12 [26].

In a word, the central element of the molecular switch of nuclear 1,25(OH)**_2_**D**_3_** signaling is VDR LBD, which can be stabilized by 1,25(OH)**_2_**D**_3_** or its analogues into agonistic, antagonistic, or inverse agonistic conformations. The positioning of H12 of the LBD is the core determinant for these conformations, as it determines the distance for the charge clamp between amino acids K246 (H3) and E420 (H12) that are essential for VDR–CoA interaction. In clinic, mutation in the VDR (E420K) may disrupt CoA binding to the VDR and causes HVDRR.

The AF-2 core sequence is highly conserved throughout the NR superfamily and it plays an important role in the ligand-dependent signal activation or repression. In fact, alanine-scanning mutagenesis shows the mutation of the last five amino acid residues GNEIS of H12 (residues 423–427) and shows no effect on binding of 1,25(OH)**_2_**D**_3_** to LBP, and its function is providing an interface for CoA or CoR proteins [27].

The amino acid glycine 423 (G423) is critical for ZK159222, partial VDR antagonist, either for the activity of the natural VDR agonist, 1,25(OH)**_2_**D**_3_** or for ZK168281, a low agonist structural derivative of ZK159222. There is a kink at amino acid residue G423 that provides the last four amino acid residues of H12, with a modulatory role for ZK159222 [28].

The AF-2 domain serves as a central motif through which CoA proteins such as SRC-1 and glucocorticoid receptor interacting protein 1 (GRIP 1) interact in a ligand-dependent manner with VDR. The major consequences of a ligand-induced conformational change of the VDR LBD make a repositioning of the AF-2 domain that together with H3 and H5 providing an interface for the interaction with CoAs that further contact components of the transcriptional machinery, which enhance transcription of NR target genes [29]. The AF-2 domain also provides an interface for the interaction of VDR LBD with a co-repressor protein that mediates the signal “repression” to the basal transcriptional machinery [30].

### 4.2. The Second Distinct Activation Domain in the VDR LBD

The second distinct activation domain in the VDR LBD is situated between D195 and I238; this domain plays an important role in 1,25(OH)**_2_**D**_3_**-activated transcription and interacts with limiting cellular factors important for VDR-activated transcription. This minimal transactivation domain (D195-I238) has a relative abundance of acidic residues (10 out of 44 residues or 23%). The NH_2_-terminal part (D195-Q223) of D195-I238 is situated within the Ω loop between H2 and H3, and the COOH- terminal part (L224-I238) contains the NH2-terminal part of H3 (L224–K 246) [31].

Like RXR, VDR LBD has a long, unordered Ω loop structure between H2 and H3. Within this loop are 25 amino acid residues (residues 199-223, SSSFSNLDLSEEDSDDPSVTLELSQ), only existing in VDR LBP for all NR. Removal of the loop does not markedly influence the functionality of the VDR. This loop that makes VDR unique among the NRs has not disappeared during evolution and suggests that functions connected to this loop still remain to be characterized [32].

**Figure 3:**
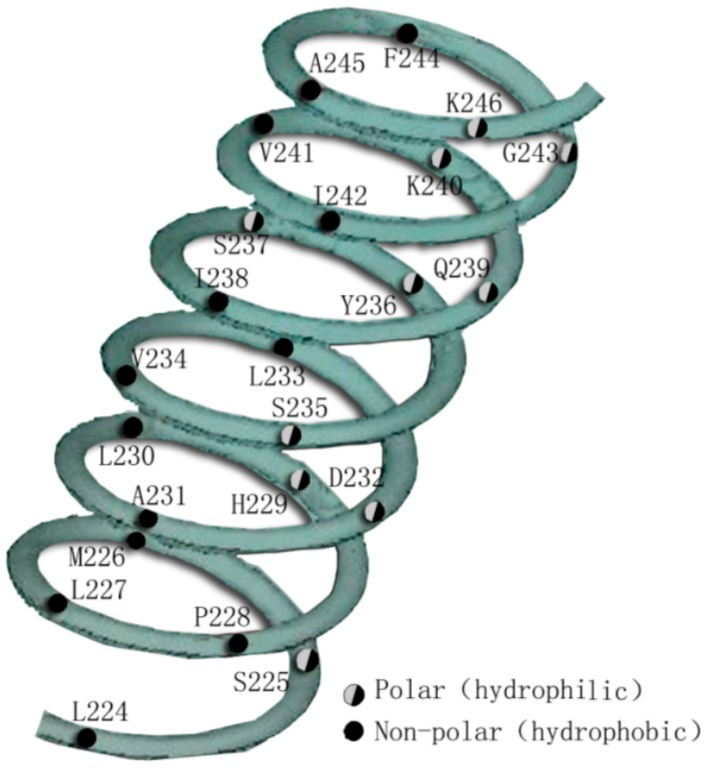
A continuous amphipathic H3 in the hVDR LBD. The polar/charged (hydrophilic) residues and non-polar residues (hydrophobic) occur on opposite faces of the helix.

There are 12 hydrophobic residues and 11 hydrophilic residues in the H3 (L224–K246). The helical wheel plot of H3 demonstrates the amphipathic α-helical nature with the distribution of hydrophobic and hydrophilic residues on opposite faces of the helix (Figure 3) [33]. There are seven hydrophobic residues (L227, L230, L233, V234, S237, I238, and K240) taking part in forming LBP, and a part of the hydrophilic residues (such as K246, H229A, D232, and Y236A) take part in forming interfaces for CoA and CoR. The H3 transactivation domain is critical for ligand-activated transcription in the full-length VDR. Mutations within H3 disrupt both transactivation by VDR and abolish p160 CoA interaction with VDR. Selection of residues within the H3 activation domain is also necessary for ligand-dependent transcription and interaction between VDR with the p160 CoA family of NR, including SRC-1 and GRIP-1 [34].

K246 at the COOH-terminus of H3 is a critical residue for transactivation. Mutation of this lysine residue to a glycine (K246G) does not affect 1,25(OH)**_2_**D**_3_** binding, VDRE interaction, or nuclear localization, but the mutant is transcriptionally silent, because the distance between K246 (H3) and E420 (H12) is essential for the VDR–CoA interaction mentioned above [24].

H229, D232, and Y236 are three residues within H3 that are determined to be important for both the autonomous activity of the minimal domain and for ligand-activated transcription of full-length VDR. Altering these amino acids individually reduces (H229A or Y236A) or abolishes (D232A or D232N) this autonomous transactivation activity. It may be a reason that these residues, especially for H229 and D232, contribute to the stability of LBD [8].

Thus, the D232 within H3 plays a vital role in the autonomous transactivation activity and its mutations in full-length VDR exhibit impaired abilities of the receptor to bind the 1,25(OH)**_2_**D**_3_** ligand or to heterodimerize with RXR in a ligand-dependent manner [8].

In addition to the AF-2 domain, Y236, located within the H3 activation domain of the VDR, is required for efficient interaction with CoAs of the SRC-1 and GRIP-1, because it may comprise a portion of the interaction surface through which p160 CoA proteins such as SRC-1 or GRIP-1 contact the VDR. On the other hand, the transcriptional enhancement observed with wild-type VDR is not apparent with mutant Y236A even in the presence of excess SRC-1 or GRIP-1. At the same time, VDR (Y236A) exhibits heterodimerization with the retinoid X receptor (RXR). Mutant analysis shows the Y236A H3 mutation bound ligand with an affinity similar to that of wild-type VDR, and both mutant Y236A and wild type VDR retain strong interaction with RXR in a ligand-dependent manner, but remain transcriptionally inactive. Mutant Y236A, in spite of retaining the ability to heterodimerize with RXR and to bind hormone, is selectively impaired in 1,25(OH)**_2_**D**_3_**-induced interaction with CoA proteins, such as SRC-1, GRIP-1, or RIP-140, both in yeast and *in vitro* binding assays. These findings demonstrate that the Y236A mutation does not influence the ability of VDR to heterodimerize with RXR or to bind ligand, as alteration of this single tyrosine residue selectively impairs both 1,25(OH)**_2_**D**_3_**-dependent interaction with the AF-2 CoAs SRC-1, GRIP-1 and RIP-140 and the subsequent transcriptional regulatory effects of the VDR [34].

The H229A mutation in full-length VDR exhibits impaired abilities to bind the 1,25(OH)_2_D_3_ ligand and to heterodimerize with RXR. Thus, the H229A mutations are inactive in hormone-mediated VDR transcription [8]. Except for this, H3 may play a role in mediating interactions between NRs and AF-2-independent CoAs such as TRAM-1 and NCoA-62 [35].

### 4.3. The Coactivator Interface between H3 and H12

In the structural relationship, a packing of the AF-2 helix onto the LBD is adjacent to H3 and H4. The function of H3 and H12 (the AF-2 domain) is to form a common transactivation surface, and requires both helices for efficient CoA interaction and transactivation. Thus, mutations in two separate distinct helices (*i.e.*, H12 and H3) disrupt 1,25(OH)**_2_**D**_3_**-induced interaction with p160 CoAs. Ligand binding forming the charge clamp structure between E420 (H12) and K246 (H3) provides a positioning for contacting the LXXLL motif of the VDR interaction box of CoA proteins mentioned above, and is just situated in the cleft among H3, H4, and H12, when the spatial structure of VDR LBD is observed [3]. The formation of a common p160 interaction surface on VDR is comprised, in part, of H12 (the AF-2 domain) and H3. The CoA interface between H3 and H12 is surrounded by the residues within H5 and H6 [36,37].

## 5. Hydrogen Bonds Trigger the Conformational Change of VDR LBD

Hydrogen bonds between three hydroxyl groups of a ligand and six polar residues of VDR LBP change the conformation of the VDR LBD, and form some interfaces where the VDR interacts with the dimer partner and transcriptional co-activators.

### 5.1. Hydrogen Bonds between the 1α-OH Group and S237 (H3) and R274 (H5) Contribute to Form a Dimer Partner and AF-2 Surface

These bonds make V234 and I238 (H3) and I271 (H4) link with each other directly; moreover, an important loop between I238 (H3) and I271 (H4) is generated. Then, the change of I271 (H5) influences its neighbor E269 (H5) and the latter interacts directly with L390 (H10/11) and R391 (H10/11) which contacts D342 (just behind H8). D342 is one of key points between RXR and VDR. Except for these residues, the substitutions of F279A (just behind H5), and H397A (H11) completely prevent VDR from heterodimerizing, and the substitutions of H229A (H3), D232A (H3), and Y295A (H6) clearly have a negative influence on heterodimerization [38]. In fact, the residues of the signature region (between V241-L263) constitute most of this loop. The loop makes L417 (H12) become fixed to I238 (H3), and F422 (H12) to H397 (H11) and Y401 (H11) to complete the AF-2 surface. Except for this, the ligand has a Van Der Waal’s contact at C-26 with V418 (H12) to stabilize the active conformation from inside. The link between F422 (H12) and H397 (H11) is a key to the formation of the AF-2 surface because it creates a basis for a charge clamp structure between E420 (H12) and K246 (H3) [24,39,40].

### 5.2. Hydrogen Bonds between the 25-OH Group and H305 and H397

The positioning of H11 is important because it forms the active conformation with a pair of residues H305 (just in front of H7) and H397 (H11) linked to the 25-OH group of 1,25(OH)**_2_**D**_3_** by hydrogen bond, and V300 (H6) to C(12) by Van Der Waal’s interaction, bottoms up the LBP at H6 to loop 6-7, and further links to residue (F422) on H12. These help H397 to make a tight contact with F422 and both residues to interact with I268 (H4/5) and Y401 (H11) [41].

### 5.3. Hydrogen Bonds between the 3-OH Group and Y143 and S278

The function of the 3-OH of ligand is less important than that of the 1-OH and the 25-OH. There are some hydrogen bonds between the Y143 3-OH group and Y143 and S278 of VDR, but residue Y143 is more important for receptor function than residue S278. In fact, the A-ring 3-OH group also docks next to C288 in the binding pocket and plays a crucial role for the contiguous segment between M284 and C288 [1].

## 6. Binding Interface of VDR LBD to CoA, CoR, and TAF

There are two classes of CoAs that may bind to VDR LBD with agonist stimulation. The first class is the histone acetylases (HATs) complex that includes PCAF and CREB-binding protein (CBP)/p300, as well as an emerging number of related factors, collectively called the p160/steroid receptor CoA (SRC) family according to their molecular weight, such as SRC-1/NCoA-1, SRC-2/GRIP1/TIF2/NCoA-2, and SRC-3/ACTR/pCIP/AIB1/RAC3 in which TIF2 can respond perfectly to 1,25(OH)**_2_**D**_3_**. The interaction between ligand-VDR LBD and the p160/SRC CoAs is mediated by a small α-helical motif containing the LXXLL sequence (where L is leucine and X is any amino acid). Ligand binding leads to realignment of H12, so it creates a basis for a charge clamp structure between E420 (H12) and K246 (H3) and reveals a hydrophobic groove between H3 and H12, where the LXXLL motifs bind [42]. Then, these CoA proteins interact with HATs, which are enzymes that decrease the positive charge of histones, and thus, the affinity of nucleosomes for DNA is maintained [43]. In this way, ligand-bound VDR can trigger to slack chromatin in the vicinity of VDREs. Another class of CoAs is the VDR-interacting protein (DRIP) complex that includes 15 members bridging the basal transcription machinery, and finally, results in activation of transcription. As a key subunit of the DRIP complex, DRIP205 interacts directly with VDR in response to an agonist, and anchors the other 14 DRIP subunits to the VDR LBD [44]. In contrast to the p160 family of CoAs, the DRIP205 and the other DRIP subunits are devoid of any intrinsic HAT activity. There are two LXXLL NR interaction motifs in the DRIP205 that we have termed NR1 and NR2. In the context of a DNA bound VDR-RXR heterodimer, NR1 of DRIP205 is required for RXR, and NR2 only for the AF-2 subdomain of VDR in response to ligand binding [45]. Several AF-2 point mutants in VDR demonstrate that the DRIP complex shares a similar sequence, and perhaps, structural requirements for VDR LBD to interact with the SRC-1/p160 family. Thus, different CoAs with distinct functions may bind to the same transactivation region of VDR LBD. Ligand-bound VDR seems to switch rapidly between the two CoA types, so that both openings of chromatin and transactivation are achieved [46].

There are three classes of CoRs possessing a binding ability to VDR LBD. CoRs are composed of several proteins, including a silencing mediator for retinoid and thyroid hormone receptors (SMRT), Alien, and NR co-repressor (NCoR). SMRT includes two SMRT silencing domains, SD-I and SD-II at the N-terminal end, and is thought to be involved in transcriptional repression and the two receptor interaction domains (RID-1 and -2) at the C-terminal end. SMRT is directly involved in the VDR-mediated repression via an RID1-specific interaction with the VDR/antagonist and a salt bridge between the RID1 of SMRT and a conserved lysine (K264) in H4 of the VDR which is central to this interaction. The VDR H12 is stabilized by direct contacts with residues of the SMRT and actively regulates the ID1 predilection of the VDR by impeding ID2-VDR association, because specific amino acid residues within the extended helix motif of SMRT-ID2 are required for VDRΔAF2 binding and deletion of H12 from VDR (VDRΔAF2) to convert it to a more effective repressor through additional interaction between SMRT-ID2 and VDR [47]. Alien, a highly conserved protein, enhances a variety of nuclear hormone receptor-mediated transcriptional silencing, including the VDR, and possesses an intrinsic transcriptional silencing function, in part, by a histone deacetylase (HDAC) dependent. The unliganded VDR directly associates with Alien, which then enhances the activity of nucleosome assembly protein 1 (NAP1)-mediated nucleosome assembly. After deposition, Alien can prevent histone displacement and drive the balance toward a more repressed chromatin state through inhibiting the histones H3 and H4 to NAP1 [48]. Alien is independent of the VDR AF-2, and interacts with the hinge region rather than with the LBD of VDR [49]. NCoR was confirmed to contact the VDR directly with similar binding affinity with both of its NR interaction domains and for its interaction with NRs, the so-called CoR-box, a conserved AAs sequence within H1 of the LBD. Thus, SMRT, Alien, and NCoR are using different interaction interfaces within the VDR. These proteins recruit DNA DnmT1, DnmT3a (DNA methyltransferases) and HDAC1, which cause chromatin condensation and transcriptional repression [50]. Other views about CoRs of VDR LBD also are being certified continuously. For example, only SMRT and Alien but not NCoR1 are related to VDR repression, and CoRs binding are less well characterized but appear to overlap that of CoAs in H3 and H5, a region blocked by H12 in the presence of ligand [51].

hTAF_II_ 28 also links to VDR LBD. The human TATA-binding protein-associated factor 28 (hTAF_II_ 28) interacts with two independent regions of the VDR LBD, one of these located between amino acids V234 and R274 spanning H3–H5 and containing the NR signature (between amino acids Y241 and L263 in the VDR LBD), and another located in H8. Interaction between hTAF_II_ 28 and the VDR LBD is ligand-reversible. The determinants for interaction with the evolutionary conserved C-terminal domain of hTAF_II_ 28, which contains the histone fold motif, are located in H3. In fact, a triply-mutated VDR [Q239F (H3), K240T (H3), S256L (H5)] does not abolish interaction with hTAF_II_ 28, as expected from the fact that hTAF_II_ 28 also interacts with the VDR H8 region. However, the interaction with the mutated VDR LBD is no longer ligand-reversible and is unable to activate transcription. The determinants of hTAFII55 and hTAF_II_ 28, for their respective interactions, may not be identical and the key domain needs to be certified [52].

## 7. Conclusions and Perspectives

The function of VDR LBD is related with ligand binding, nuclear localization, forming different surfaces for dimerization and interaction with CoA and CoR proteins [53]. VDR as other NRs show some adaptability of the LBP upon binding to diverse ligands such as PPAR, TR, EcR, ER and so on. At the same time, the amount of ligand is similar to that observed for other NRs. VDR as other NRs also shows a common mechanism of ligand-triggered conformational change [54]. Functional diversity is determined by its LBP structure and ligands flexibility [55]. Forming interfaces for dimerization, CoA and CoR proteins are closely related with the composition and properties of amino acids of H3 and H12 [56]. Therefore, 36 amino acid residues that line the LBP and six of these residues that have hydrogen-bonds linking with ligand are important catering for ligands binding [57]. In the 1,25(OH)**_2_**D**_3_** signaling, H3 and H12 play an important role in the course of conformational change resulting in the provision of interfaces for dimerization, CoA, CoR, and hTAF_II_ 28. In summary, this paper provides a detailed description of the amino acid residues stabilizing the structure and taking part in conformational change of VDR LBD according to functional domains.
